# High‐Performance Flexible Pressure Sensor with a Self‐Healing Function for Tactile Feedback

**DOI:** 10.1002/advs.202200507

**Published:** 2022-04-15

**Authors:** Mei Yang, Yongfa Cheng, Yang Yue, Yu Chen, Han Gao, Lei Li, Bin Cai, Weijie Liu, Ziyu Wang, Haizhong Guo, Nishuang Liu, Yihua Gao

**Affiliations:** ^1^ Key Laboratory of Material Physics, Ministry of Education, School of Physics and Microelectronics Zhengzhou University Zhengzhou 450052 P. R. China; ^2^ Center for Nanoscale Characterization and Devices (CNCD) School of Physics and Wuhan National Laboratory for Optoelectronics (WNLO) Huazhong University of Science and Technology (HUST) Wuhan 430074 P. R. China; ^3^ Information Materials and Intelligent Sensing Laboratory of Anhui Province Key Laboratory of Structure and Functional Regulation of Hybrid Materials of Ministry of Education Institutes of Physical Science and Information Technology Anhui University Hefei 230601 P. R. China; ^4^ The Institute of Technological Sciences Wuhan University Wuhan 430072 P. R. China; ^5^ Collaborative Innovation Center of Light Manipulations and Applications Shandong Normal University Jinan 250358 P. R. China

**Keywords:** flexible piezoresistive sensors, low‐cost, self‐healing, tactile feedback, universality

## Abstract

High‐performance flexible pressure sensors have attracted a great deal of attention, owing to its potential applications such as human activity monitoring, man–machine interaction, and robotics. However, most high‐performance flexible pressure sensors are complex and costly to manufacture. These sensors cannot be repaired after external mechanical damage and lack of tactile feedback applications. Herein, a high‐performance flexible pressure sensor based on MXene/polyurethane (PU)/interdigital electrodes is fabricated by using a low‐cost and universal spray method. The sprayed MXene on the spinosum structure PU and other arbitrary flexible substrates (represented by polyimide and membrane filter) act as the sensitive layer and the interdigital electrodes, respectively. The sensor shows an ultrahigh sensitivity (up to 509.8 kPa^–1^), extremely fast response speed (67.3 ms), recovery speed (44.8 ms), and good stability (10 000 cycles) due to the interaction between the sensitive layer and the interdigital electrodes. In addition, the hydrogen bond of PU endows the device with the self‐healing function. The sensor can also be integrated with a circuit, which can realize tactile feedback function. This MXene‐based high‐performance pressure sensor, along with its designing/fabrication, is expected to be widely used in human activity detection, electronic skin, intelligent robots, and many other aspects.

## Graphical abstract

A MXene‐based high‐performance flexible piezoresistive sensor is fabricated by a simple spraying method. Due to the abundant hydrogen bonds of polyurethane, the pressure sensor owns a self‐healing function. By integrating the flexible piezoresistive sensor with manipulator, resistance–voltage converter, SGS‐THOMSON Microelectronics (STM) 32 microprogrammed control unit signal analysis unit, and touchscreen, a tactile feedback system is achieved.

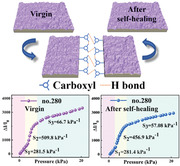

## 1 Introduction

The rapid growth of bioelectronics,^[^ ^1^, ^2^ ^]^ smart home,^[^ ^3^ ^]^ intelligent robots,^[^ ^4^ ^]^ and human–machine interfaces^[^ ^5^, ^6^ ^]^ has greatly promoted the market demand for flexible pressure sensors. Flexible pressure sensors are mainly based on several types of working mechanisms, such as capacitive,^[^ ^7^, ^8^ ^]^ piezoresistive,^[^ ^9^, ^10^, ^11^ ^]^ piezoelectric,^[^ ^12^, ^13^ ^]^ and triboelectric.^[^ ^14^ ^]^ Piezoresistive sensors based on piezoresistive effect are used due to their high sensitivity, fast signal response, simple manufacturing process, and stable sensing performance.^[^ ^15^ ^]^ Accordingly, the preparation of high‐performance piezoresistive sensors that can meet the needs of various situations has become a hot topic in recent years. The recent research on the design of flexible piezoresistive sensors mainly focuses on the microstructural design of the sensitive layers. It has been proved that the microstructural design of the flexible substrate can significantly improve the sensing performance of the sensor.^[^ ^16^ ^]^ Some microstructures have received special attention because of their simple preparation processes, the most typical of which is the spinosum structure made from sandpaper.^[^ ^10^, ^17^ ^]^ Although some progress has been made in the research of piezoresistive sensors on microstructure, the ideal flexible piezoresistive sensor only has excellent sensing performance, which is far from enough. In the practical applications, the flexible piezoresistive sensors unavoidably face complex working environments, and the sensitive layer is easily subjected to a variety of external mechanical damage. Moreover, most fabrication processes of flexible sensors limit the choice of flexible substrates. All these problems hinder the wide application of flexible piezoresistive sensors.^[^ ^18^ ^]^

MXene, as a 2D sheet material, can be easily combined with other flexible substrate materials to form sensitive layers, and some MXene based sensitive layers with microstructure can be obtained, which can effectively improve the performance of the sensor.^[^ ^19^ ^]^ The flexible substrate materials for the flexible piezoresistive sensors mainly include polyimide (PI),^[^ ^20^, ^21^ ^]^ polydimethylsiloxane,^[^ ^20^, ^22^ ^]^ and polyethylene terephthalate.^[^ ^23^, ^24^ ^]^ Self‐healing materials have attracted the attention of researchers due to their ability to provide long working life and excellent mechanical stability to sensors.^[^ ^25^ ^]^ Polyurethane (PU) can be healed by itself due to its hydrogen bond, which can promote wide application prospects in the research and development of various flexible electronic devices.^[^ ^26^ ^]^

The diverse application scenarios of the flexible piezoresistive sensors raise various requirements for the flexible substrate materials of electrodes. Au, Ag, and indium tin oxide,^[^ ^27^, ^28^, ^29^ ^]^ as traditional electrode materials, have excellent conductivity. However, the preparation process of these electrodes is still dominated by the traditional methods such as magnetron sputtering, vacuum evaporation, and pulse electrochemical deposition.^[^ ^30^, ^31^ ^]^ These technologies not only increase costs of the sensor preparation but also limit the choice of the flexible substrate. For example, the vacuum evaporation requires the high temperature heating, which results in the demand on the heat bearing capacity of the substrate.^[^ ^32^ ^]^ Moreover, the rigidity of metal and metal oxide will results in cracks in the electrode when the substrate is bent, which will seriously affect the robustness of the sensors. Therefore, a simple, low‐cost, and universal method for preparing the flexible electrodes with high flexibility and conductivity must be established.

The ultimate goal of manufacturing the flexible piezoresistive sensor is for practical application. Tactile feedback is an essential function for the practical application of sensors.^[^ ^33^ ^]^ Touch can be felt all over the body, helping people feel pressure. Tactile feedback can make corresponding instructions according to the pressure felt by the human body to ensure its normal activities. Therefore, the tactile feedback is very important for people. The right amount of force is strictly needed for grasping an object, especially tiny and fragile ones. The body feels the pressure and passes it on to our brain, which gives us tactile feedback so that we can smoothly grab an object.^[^ ^34^ ^]^ We must give the robot tactile perception and feedback functions to make it work like a human being. The tactile feedback of the sensor can be achieved by combining the sensors with a circuit. However, tactile feedback is rarely mentioned in most of the work related to the flexible piezoresistive sensors.

Herein, we propose an MXene‐based high‐performance flexible piezoresistive sensor with self‐healing function for tactile feedback by a simple full spray method. PU with a spinosum structure is used as the flexible substrate of the sensitive layer. The MXene/PU sensitive layer with the self‐healing function was obtained by depositing the MXene nanosheets on the spinosum structure surface of PU. Furthermore, the MXene‐based flexible interdigital electrodes were prepared by a low‐cost and universal template spraying method. The choice of the flexible substrate for the interdigital electrodes prepared by this method can be arbitrary. We prepared the MXene‐based piezoresistive sensors with PI and membrane filter as substrates to verify the arbitrariness of the flexible substrate of the interdigital electrodes. Their sensitivity can be up to 509.8 and 408.4 kPa^–1^, the corresponding response times are 67.3 and 68.4 ms and the recovery times are 44.8 and 46.5 ms. Moreover, the current of the sensor remains stable after 10 000 pressure cycles. In addition, the sensitivity of the sensor (PI as the flexible substrate for the interdigital electrodes) can still reach up to 456.9 kPa^–1^ after the sensitive layer experienced “cutting‐healing” for 18 h. In practical application, the sensor can not only detect human activities but also combine with software and hardware design to make the manipulator realize tactile feedback function. Therefore, the fully sprayed MXene‐based high‐performance pressure sensor has great potential in human activity detection, electronic skin, intelligent robots, and so on.

## 2 Results and Discussion

### 2.1 Fabrication and Characterization

**Figure 1:**
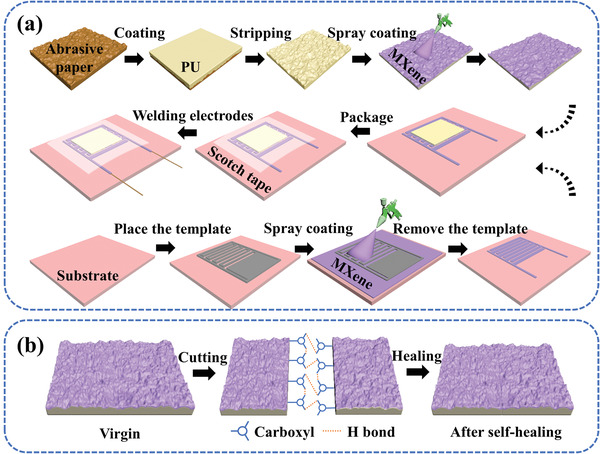
a) Fabrication procedure of the fully sprayed MXene‐based high‐performance pressure sensor. b) Schematic diagram of the self‐healing mechanism of the sensitive layer.

The fabrication procedure of the fully sprayed MXene‐based high‐performance pressure sensor is shown in **Figure** **1**a. The preparation of the sensor includes two important procedures. One is the preparation of the sensitive layer. The flexible substrate with random distribution spinosum structure can be obtained by casting PU on the microstructure surface of an abrasive paper. After drying and stripping, the MXene conductive layer was evenly distributed throughout the spinosum structure surface of the flexible substrate, which formed the spinosum MXene/PU sensitive layer. A series of abrasive papers with different roughness such as nos. 100, 180, 280, 400, 600, and 800 were prepared to further study the influence of the spinosum MXene/PU sensitive layer on the sensor. The other one is the preparation of the MXene‐based interdigital electrodes. An arbitrary flexible film (represented by PI and membrane filter) was selected as the substrate of the interdigital electrodes. The interdigital electrodes mask plate was placed on the substrate, and a certain amount of MXene solution was sprayed on the surface of the substrate. After removing the interdigital electrodes mask plate, the MXene‐based interdigital electrodes with a thickness of ≈5 µm can be obtained. Finally, the spinosum MXene/PU sensitive layer and MXene‐based interdigital electrodes were assembled together, and the MXene‐based high‐performance pressure sensor was obtained.

The PU shows excellent self‐healing characteristics. Figure 1b shows the cutting‐healing process of the sensitive layer with spinosum structure. After cutting the sensitive layer with a scalpel, the sensitive layer will complete self‐healing under the action of a large number of hydrogen bonds at the PU interface. Stretchability and compressive strength tests on PU before and after self‐healing were performed. Figure S1a in the Supporting Information shows the stress–strain curve of PU in virgin and after self‐healing state. The strain of the original PU can reach 1600%, while that of the self‐healing PU still attains more than 1400%. The pressure–strain curve in Figure S1b in the Supporting Information shows that PU has excellent compressive performance, the compressive performance does not significantly weaken after self‐healing. Therefore, PU has excellent stretchability, compression resistance, and self‐healing ability. These features ensure that the sensor has excellent mechanical properties and robustness.

**Figure 2:**
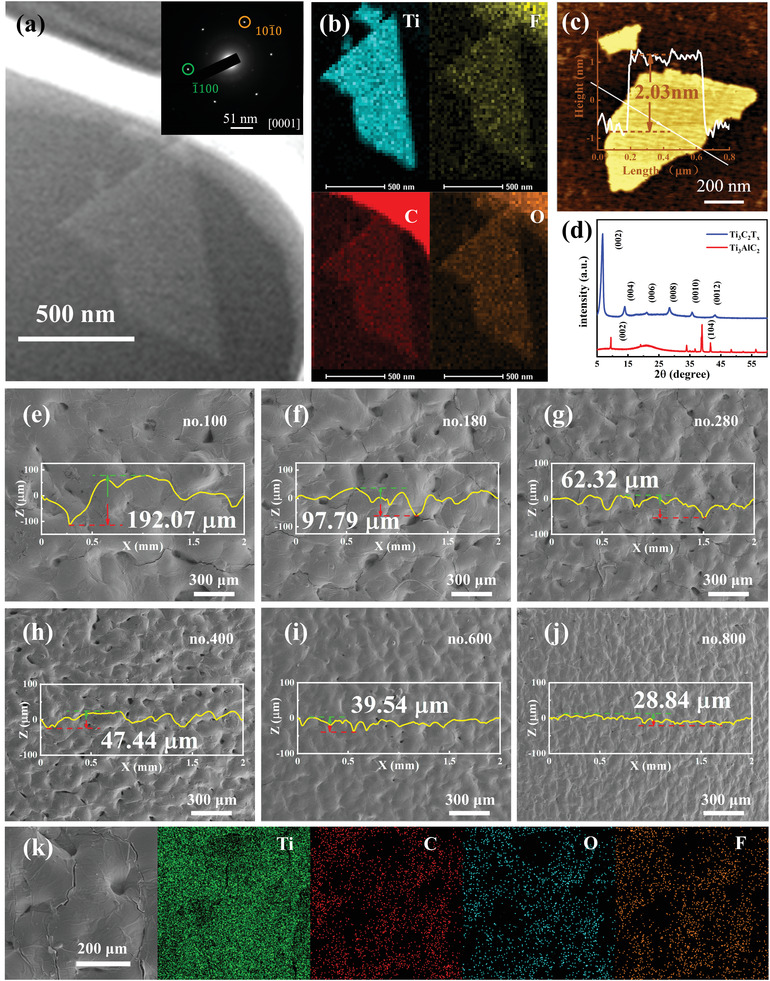
a) TEM image of the MXene nanosheets, and the inset is the corresponding SAED pattern. b) energy dispersive spectrometer (EDS) mapping images of MXene (Ti_3_C_2_T*_x_*). c) AFM image of the MXene nanosheet. d) XRD pattern of the MAX phase (Ti_3_AlC_2_) and MXene Ti_3_C_2_T*_x_* nanosheet. e–j) SEM images of the sensitive layers with the different spinosum structures (nos.100, 180, 280, 400, 600, and 800). k) EDX mapping images of no. 280 sensitive layer.

MXene, which owns more adjustable functional groups (—F, —H, —OH),^[^ ^35^ ^]^ good hydrophilicity,^[^ ^36^, ^37^ ^]^ high conductivity (6500 S cm^–1^),^[^ ^38^, ^39^ ^]^ and excellent mechanical properties,^[^ ^40^, ^41^, ^42^ ^]^ was used as sensitive material.^[^ ^43^ ^]^ The preparation of MXene nanosheets is shown in Figure S2 in the Supporting Information and the mixed solution of HCl and LiF was used to etch the “Al” layer from the precursor MAX phase (Ti_3_AlC_2_). After the etching, the multilayer MXene (Ti_3_C_2_T*_x_*) was achieved and the single‐layer MXene nanosheets were obtained by ultrasonic stripping.^[^ ^44^ ^]^ The collected final product is a dark green colloidal dispersion, in which MXene nanosheets are evenly dispersed. When the laser is irradiated in the MXene solution, Tyndall effect will be evident (Figure S3a, Supporting Information).^[^ ^45^ ^]^ Furthermore, the Raman spectrum also indicates the successful synthesis of MXene (Figure S3b, Supporting Information). The Ti—C and C—C vibrations of the oxygen terminated MXene cause characteristic peaks at ≈203 and 724 cm^–1^, respectively. In addition, the vibrations of the oxygen atoms (E_g_ and A_1g_, respectively) causes characteristic peaks at ≈386 and 571 cm^–1^.^[^ ^46^ ^]^ We characterized MXene nanosheets by transmission electron microscopy (TEM), as shown in **Figure** **2**a. The image inserted in the upper right corner is the corresponding selected area electron diffraction (SAED), from which the MXene nanosheets show a hexagonal crystal structure. Figure 2b is the corresponding energy‐dispersive spectroscopy (EDS) mapping images of the MXene nanosheets. The C, Ti, O, and F elements are uniformly distributed throughout the Ti_3_C_2_T*_x_* nanosheets. Figure 2c shows the thickness (2.03 nm) of the specified MXene nanosheets measured with an atomic force microscope (AFM), which proves that the single layer MXene was successfully prepared. The MAX phase and MXene were characterized by X‐ray diffraction (XRD), as shown in Figure 2d. After the synthesis of MXene, the peak of the MAX phase at ≈39° disappears, which indicates that the “Al” layer in the precursor was etched. In addition, the peak value (002) of the MAX phase is around 10°, while the peak value (002) of MXene shifts to the left at ≈6°, indicating that the interlayer spacing increases.^[^ ^47^, ^48^ ^]^ All these phenomena indicate the successful synthesis of the MXene nanosheets.

Figure 2e–j shows the sensitive layer (denoted as nos. 100, 180, 280, 400, 600, and 800) images with the different spinosum structures on the PU characterized by scanning electron microscopy (SEM). These spinosum structures come from the abrasive papers with the different roughness and the surface curves of the spinosum structure were measured with a step profiler with a distance of 2.0 mm. These figures exhibit that the density of spinosum in the same area increases from no.100 to no.800, while the maximum height of spinosum decreases in turn. Figure 2k shows the EDS mapping images of the spinosum structure surface of the no.280 sensitive layer, which proves that MXene has been uniformly and densely sprayed on the spinosum structure surface. In addition, the square resistance of the spinosum structure sensitive layer with the increase of spraying times was summarized (Figure S4, Supporting Information). All structures show a similar trend of change. The resistance quickly drops in the early stages and slowly later. Moreover, the resistance rapidly decreases with the gradual flattening of the spinosum structure (from no.280 to no.600).

The electrode was also carefully selected as an important part of the pressure sensor. The interdigital electrodes are widely used in sensors because of their ease of operation. The time‐dependent line resistance of the interdigital electrodes was measured during the preparation of the MXene‐based interdigital electrodes (Figure S5a, Supporting Information). The line resistance less than 5.0 Ω means that the MXene‐based interdigital electrodes have been successfully prepared. The successful preparation of the interdigital electrodes is attributed to the good hydrophilicity and conductivity of MXene. Figure S5b in the Supporting Information depicts the optical image of interdigital electrodes on the PI substrate. Figure S5c in the Supporting Information reveals the thickness of the interdigital electrodes, which is ≈5.245 µm. The small resistance can guarantee that the circuit is smoothly energized and the appropriate thickness of the interdigital electrodes will provide more microstructures. The above‐mentioned two aspects are beneficial to the improvement of the sensitivity of the sensor. Figure S6a–c in the Supporting Information shows the optical images of the MXene/PU sensitive layer, MXene‐based interdigital electrodes (PI as flexible substrate), and MXene‐based flexible pressure sensor. Moreover, bending tests were carried out on the different components and they all show excellent flexibility. Therefore, the sensor can be attached to a variety of complex 3D carrier surfaces.

### 2.2 Sensing Properties of the MXene‐Based Pressure Sensor

The flexible pressure sensor, as a “medium,” converts the pressure signal into an electrical signal, which makes the pressure digital and visual. Some performance parameters are needed to quantitatively evaluate the comprehensive performance of the device to design a flexible pressure sensor with excellent comprehensive performance and strong practicability. These performance parameters include sensitivity (S), sensing range, response time, recovery time, and cycle stability.^[^ ^36^ ^]^ Here, we study the sensing performance of the sensor by using the system assembled by a computer, motion propulsion device, dynamometer, and high‐precision electric signal detection source meter (Agilent B2901A). When the driving voltage was 0.10 V, the change of the real‐time output electric signal of the sensor was measured by the testing system. S refers to the slope of the curve of resistance versus applied pressure and it is a key performance parameter describing the ability of the pressure sensor to convert pressure signal into resistance signal. The sensitivity of a piezoresistive sensor is defined as follows: S  =  *δ*(ΔI/I_0_)/*δ*P, where ΔI is the current change value under external force, *I*_0_ is the current value in the initial state, and P is the external pressure.^[^ ^49^ ^]^

**Figure 3:**
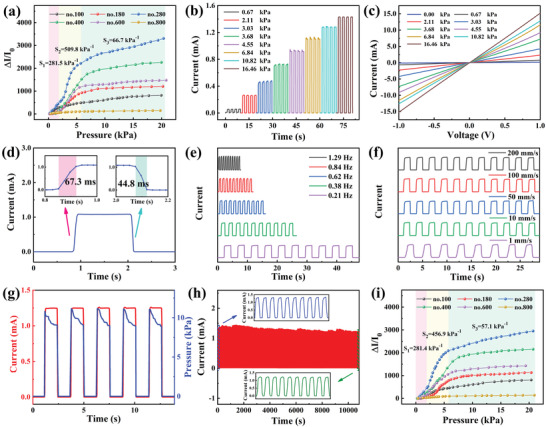
Sensing properties of the MXene‐based pressure sensor (PI as the interdigital electrodes substrate). a) Sensitivity of the sensors from no.100 to no.800. b) The *I–T* curves of the no.280 sensor under serial pressures. c) The *I–V* curves of the no.280 sensor under serial pressures. d) The response and recovery time of the sensor. e) The frequency response performance of the sensors under 1.52 kPa. f) The different speed response performances of the sensors under 1.81 kPa. g) The response of *I–T* and *P–T* curves under periodic loading–unloading cycles. h) After 10 000 pressure cycles at 14.2 kPa, the device shows excellent stability. i) Sensing sensitivities of the nos.100–800 sensors were detected after the sensitive layer self‐healing for 18 h.

The sensitivities of the fully sprayed MXene‐based sensors (PI as flexible substrate) were measured from no.100 to no.800, and the sensor of no.280 has the highest value compared with the others, which is attributed to the interaction mechanism between the sensitive layer and the interdigital electrodes, as shown in **Figure** **3**a. The sensitivity could be divided into three sections: *S*_1_ is located in the low‐pressure range (0.20–1.70 kPa), *S*_2_ is in the middle‐pressure range (1.70–5.70 kPa), and *S*_3_ exists in the high‐pressure range (5.70–20.30 kPa). The corresponding values of *S*_1_, *S*_2_, and *S*_3_ are 281.54, 509.78, and 66.68 kPa^–1^, respectively. The following investigations on other properties were performed by the no.280 sensor. The current–time (*I–T*) curves show a gradual increasing trend in the current with the gradual increase of the external pressures (Figure 3b). Figure 3c shows the current–voltage (*I–V*) curves of the sensor with the voltage varying from −1.0 to 1.0 V, and the linear dependence of the voltage on the current at various external pressures indicates a good ohmic contact between the sensitive layer and the interdigital electrodes. Furthermore, the sensor exhibits a rapid response (67.8 ms) and recovery time (44.8 ms) due to the excellent resilience of the PU (Figure 3d). Figure 3e,f shows that the flexible piezoresistive sensor can identify the external pressures (1.52 and 1.81 kPa) from the different frequencies and speeds. Figure 3g reveals the *I–T* and pressure–time (*P–T*) curves under the same periodic pressure. The two curves are highly synchronized, which further proves the quick response time and excellent performance of the piezoresistive sensor. The 10 000 cycles of loading and unloading were also tested to further evaluate the service life and mechanical stability of the piezoresistive sensor (Figure 3h). During the loading and unloading, the output current signal of the sensor remains stable. After 10 000 cycles, the attenuation of the electrical signal is negligible. Accordingly, the sensor has excellent stability and durability under normal conditions. Table S1 in the Supporting Information shows the sensing performance of this sensor and other MXene‐based piezoresistive sensors, indicating that every performance index of the sensor is excellent.

Stretching and compression simultaneously occur when the sensor is bent by an external force. Here, the sensing performance of the sensor in the bending states was tested. The test system, which is used to test the sensor at different bending angles, is shown in Figure S7a in the Supporting Information. The *I–T* curve of the different bending angles is shown in Figure S7b in the Supporting Information, and the current intensity of the sensor increases with the increase of the bending angle. Figure S7c in the Supporting Information shows the *I–T* curve of the sensor stretching between 0° and 90°. These findings prove that the sensor can also detect stretching and compression caused by bending.

However, the sensor would inevitably suffer from the external mechanical damage during practical application. Consequently, a sensor with a self‐healing function that can improve the durability of the sensor in practical application must be prepared. As previously mentioned, the fully sprayed MXene‐based pressure sensor has the self‐healing function. Figure S8a–d in the Supporting Information shows the whole cutting‐healing process of the sensor sensitive layer in the natural environment. After 18 h of the self‐healing, the sensitive layers are closely integrated again, the MXene conductive layers are tightly in contact with each other, the sensitivities of the nos.100–800 sensors were tested (Figure 3i) and the no.280 sensor still shows the highest sensitivity, as shown in Figure S8d in the Supporting Information. The sensitivities in the low‐pressure range (0.30–2.00 kPa), middle‐pressure range (2.00–5.70 kPa), and high‐pressure range (5.70–20.70 kPa) are 281.4, 456.9, and 57.1 kPa^–1^, respectively. The data of the nos.100– 800 sensors before and after the cutting‐healing are listed in Table S2 in the Supporting Information. In comparison with the virgin state, the sensitivity of the sensors after the cutting‐healing slightly decreases. The sensitivity of the no.280 sensor reduces by only 0.04% in the low‐pressure range.

Many flexible substrates are unavailable for sensor fabrication due to the limitation of the current manufacturing process. Mixed cellulose filter membrane, as a commonly used filter membrane in laboratory, has the characteristics of super‐flexibility. However, this membrane cannot be applied to the flexible sensors as the electrode substrates due to the limitations of traditional electrode preparation technology. To prove that the preparation of interdigital electrodes by spraying is a low‐cost and universal method, a mixed cellulose filter membrane was selected as the flexible substrate of the interdigital electrodes, and the MXene‐based interdigital electrodes were prepared by the same method as above. The sensor still has excellent sensing performance, as shown in Figure S9a in the Supporting Information: *S*_1_ is in the low‐pressure range (0.36–2.34 kPa), *S*_2_ exists in the middle‐pressure range (2.34–4.57 kPa), and *S*_3_ is located in the high‐pressure range (4.57–19.73 kPa), and the corresponding values of *S*_1_, *S*_2_, and *S*_3_ are 99.8, 408.4, and 23.4 kPa^–1^, respectively. Figure S9b in the Supporting Information shows the *I–T* curve of the sensor. The output current signal of the sensor regularly increases with the increase of the pressure. Figure S9c in the Supporting Information exhibits the *I–V* curves of the sensor, which indicates that the sensor has good ohmic contact. The sensor shows fast response (68.4 ms) and recovery time (46.5 ms), as shown in Figure S9d in the Supporting Information. Moreover, the *I–T* and *P–T* curves are highly synchronized, proving once again that the sensor has extremely fast response speed (Figure S9e, Supporting Information). After 10 000 pressure cycle tests at 10.7 kPa, the sensor still maintains excellent stability (Figure S9f, Supporting Information). The excellent sensing performance of the sensor proves the universality of the spraying method.

### 2.3 Working Mechanism of the Fully Sprayed MXene‐Based Pressure Sensor

**Figure 4:**
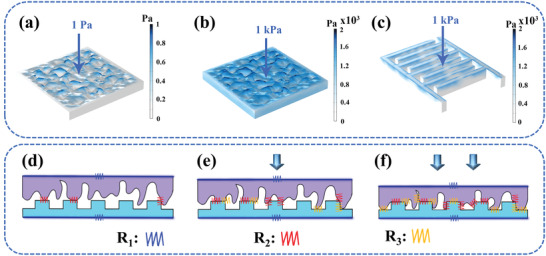
Working mechanism of the fully sprayed MXene‐based pressure sensor. Simulation results of the pressure distribution of the sensitive layer at a) 1.0 Pa and b) 1.0 kPa. c) Simulation result of the pressure distribution of the interdigital electrodes at 1.0 kPa. Schematic diagrams of circuit molds corresponding to d) initial state, e) light load state, and f) heavy load state.

A model of the spinosum structure MXene/PU sensitive layer was established according to the SEM image and surface contour curve of the sensitive layer to understand the sensing mechanism of the designed fully sprayed flexible pressure sensor (Figure 2e–j). The model of MXene interdigital electrodes was also established according to the optical image and thickness of interdigital electrodes (Figure S5b,c, Supporting Information). The meshes of the models use densely arranged free triangles (Figure S10, Supporting Information). Then the stress distribution of the two components under different pressures is analyzed by using the finite element method. The stress of the MXene/PU sensitive layer is only distributed on the tip of the spinosum structure when the external load pressure is 1.0 Pa, as shown in **Figure** **4**a. The stress distribution concentrates on the top of the spinosum structure and extends to the bottom of the spinosum structure when the external load pressure is increased to 1.0 kPa (Figure 4b). Figure 4c demonstrates that the stress is evenly distributed on the surface of the MXene‐based interdigital electrodes when the external load pressure is 1.0 kPa. These results indicate that the contact between the spinosum structure and the interdigital electrodes becomes closer with the increase in the external load pressure.

Based on the above simulation analysis, the cross‐sectional schematic of the interaction between the sensor sensitive layer and the interdigital electrodes under the initial state, light load state, and heavy load state has been created (Figure 4d–f). Moreover, an equivalent circuit has been postulated to describe the working mechanism of the sensor, as follows:$\;R\;=R_{1}\;+\frac{R_{2}R_{3}}{R_{2}+R_{3}}$; where *R*_1_ is the intrinsic resistance of the sensitive layer and the interdigital electrodes, *R*_2_ represents the contact resistance between the sensitive layer and the interdigital electrodes, and *R*_3_ represents the resistance generated by the extrusion of the spinosum structure on the surface of the sensitive layer. In the initial state, slight contact occurs between the interdigital electrodes and the interface of the sensitive layer (Figure 4d). When a light load is applied to the sensor, the contact area of the interface between the two components increases and is accompanied by a slight deformation of the spinosum structure of the sensitive layer (Figure 4e). When the contact area increased, the conductive channel and conductivity also increased. In this process, the change of resistance in the circuit is mainly attributed to *R*_1_ and *R*_2_ because the deformation degree of the spinosum structure is deficient. Next, the contact area continues to increase, and the spinosum structure of the sensitive layer is squeezed during the continuous loading process. *R*_1_, *R*_2_, and *R*_3_ all made important contributions to the reduction of resistance in the circuit (Figure 4f). When the interface contact is close to saturation and the load continues, the only change in the circuit's resistance due to the decrease of *R*_3_. Accordingly, the increase in the conductivity of the sensor will slow down. This finding is consistent with the experimental results, and the sensor shows the highest sensitivity when *R*_1_, *R*_2_, and *R*_3_ can all contribute to the change of the resistance in the circuit. In addition, this mechanism can explain that the no.280 sensor has the best sensing performance.

The spinosum structures on the surface of the sensitive layer are sparse for the no.100 sensor, so the change of *R*_2_ in the equivalent circuit is relatively small. Meanwhile, the large spinosum structures of the no.100 sensor make it difficult to deform and the change of *R*_3_ in the equivalent circuit is also limited. In the case of the no.800 sensor, the small and dense spinosum structures of the sensitive layer will not only lead to weak deformation ability but also make the contact interface difficult to expand. Accordingly, *R*_2_ and *R*_3_ will show minor changes in the equivalent circuit. However, the *R*_2_ and *R*_3_ of the no.280 sensor in the equivalent circuit change most obviously after being subjected to external pressure. Therefore, the no.280 sensor can make fully use of the spinosum structures, which allows the sensor to be more sensitive to pressure.

### 2.4 Practical Applications of the Fully Sprayed MXene‐Based Pressure Sensor

**Figure 5:**
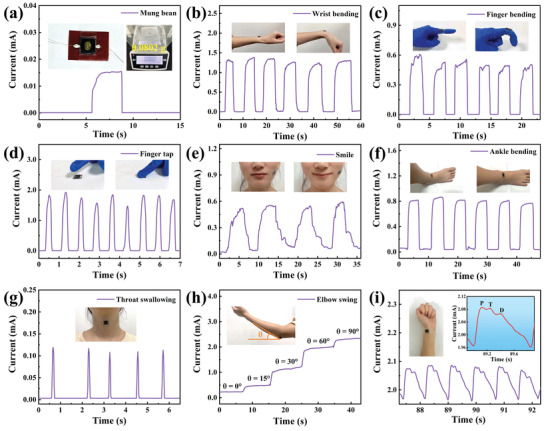
Applications of the fully sprayed MXene‐based pressure sensor in minimum detectable pressure and real‐time monitoring of human activities. The signal responses in the form of current changes come from a) tiny object pressure provided by a grain of mung bean (7.8 Pa), b) wrist bending, c) finger bending, d) finger tap, e) smile, f) ankle bending, g) throat swallowing, h) elbow bending at different angles, and i) wrist pulse (the enlarged illustration is a magnified view of the pulse vibration waveform).

The minimum detection limit of the flexible piezoresistive sensor is also considered as one of the key factors that determine the application range of the sensor. Herein, a mung bean and a soybean were used to evaluate the detection ability of the sensor under an extremely low‐pressure. The tiny pressure exerted by a mung bean (7.8 Pa) or a soybean (26.3 Pa) can be clearly detected by the sensor, as shown in **Figure** **5**a and Figure S11 in the Supporting Information. Therefore, the sensor has great potential applications in real‐time monitoring of human activities and tiny physiological signals. The sensor is attached to the muscles or joints of the human body with adhesive tape, which successfully realizes real‐time monitoring of the physical signals generated by the human activities such as wrist flexion, finger flexion, finger pressing, smiling, ankle bending, and throat swallowing (Figure 5b–g). The electrical signal output by the sensor changes with the variation of elbow bending angle, so the human behavior can be accurately inferred by the quantitative analysis of the output electrical signal, as shown in Figure 5h. Moreover, the pulse beat can be detected in real‐time by tightly sticking the sensor to the wrist artery. Figure 5i shows the regular pulse wave of ≈76 beats per minute (bpm) for a woman. The curve of pulse waves can not only obtain the bpm of people but also indicate important physiological signals such as blood pressure and arteriosclerosis. The characteristic peaks of the pulse waves include percussion (P), tidal (T), and dicrotic (D), as shown in the insert of Figure 5i. The vascular aging and arterial stiffness can be observed by the radial enhancement index (AI_r_ = *T*/*P*).^[^ ^50^, ^51^ ^]^ We also tested the practical application of the sensor after self‐healing. The sensor can still monitor human activities in real‐time, as shown in Figure S12 in the Supporting Information.

**Figure 6:**
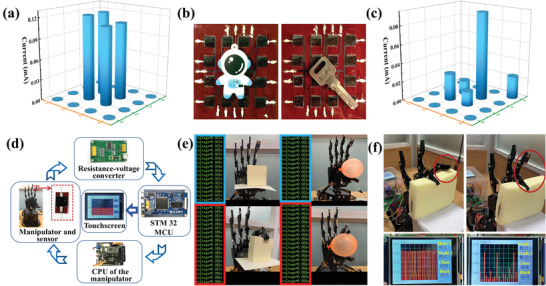
Applications of the fully sprayed MXene‐based pressure sensor in the tactile sensing. a) Related pressure distribution is obtained by the astronaut pendant on the surface of electronic skin. b) Optical images of an astronaut pendant and a key on the surface of electronic skin. c) Related pressure distribution is obtained by a key on the surface of electronic skin. d) A general schematic diagram of a pressure sensing system that enables the manipulator to realize accurate tactile feedback. e) The system is used to catch tofu and balloon. f) The mechanical palm grabs‐releases the soft bread with different frequencies.

Besides detecting various physiological signals of the human body, the sensor can also be applied to the tactile perception, such as electronic skin and robot sensing devices.^[^ ^52^, ^53^ ^]^ We also designed a 4 × 4 fully sprayed MXene‐based piezoresistive sensor array to explore its sensing ability to the pressure distribution and its application in the field of artificial electronic skin. The current intensities at the different positions of the array sensor are distinguished according to the weight of the object when an astronaut pendant and a key are placed on the array surface, as shown in **Figure** **6**a–c. This notion means that the weight or pressure distribution of a specific object can be qualitatively and quantitatively analyzed by referring to the current intensity of each matrix point.

Furthermore, the intelligent robot industry has rapidly developed in recent years. Tactile feedback is an important part of a robot's working process.^[^ ^54^ ^]^ A single tactile perception cannot realize the whole process of “action‐perception‐feedback” for robots like humans.^[^ ^34^, ^55^ ^]^ Therefore, we have designed a tactile sensing and feedback system for the mechanical palm, which can make the manipulator feel the pressure change and realize accurate tactile feedback. The system contains five parts, namely, the manipulator and sensor, resistance‐voltage converter, SGS‐THOMSON Microelectronics (STM) 32 microprogrammed control unit (MCU), central processing unit (CPU) of the manipulator, and touchscreen, as shown in Figure 6d. Figure S13a in the Supporting Information shows the hardware structure diagram of the device in operation. The designed sensor is attached to the surface of the mechanical finger, which is used to sense the pressure change when the manipulator touches the object in the process of executing the action commands. The sensor is connected to the resistance‐voltage converter and collects the change of the output electric signal of the flexible pressure sensor. Then, the data are transmitted to the STM 32 MCU for signal analysis and processing. The analyzed data are transmitted to the CPU of the manipulator to control the manipulator and make corresponding tactile feedback. The diagram of the software system in the device is shown in Figure S13b in the Supporting Information. The STM 32 MCU is also connected with a touchscreen to display the change curve of the electrical signals. In Figure 6e, the manipulator grabs fragile tofu and deformable balloon, respectively. The electrical signal output by the sensor greatly changes when the manipulator just touches the tofu and balloon. The manipulator immediately provides feedback according to the change of the electrical signal and stays on the surface of the object to protect its original shape (Videos S1 and S2, Supporting Information). Moreover, the manipulator is set to grab the soft bread at different frequencies. The soft bread maintains its original shape. We can see the change curve of the electrical signal of the pressure sensor from the touchscreen in Figure 6f, which realizes the digitization and visualization of the force. The above system completes the functions of tactile perception and feedback. Therefore, the sensor has great potential in the application for the tactile feedback of robots.

## 3 Conclusions

In summary, this work reports a simple and universal method to prepare the high‐performance pressure sensor with self‐healing function for tactile feedback. The device consists of spinosum MXene/PU and MXene‐based interdigital electrodes, and the interaction of these two parts results in an excellent performance. The sensitivity of the sensor (PI as the interdigital electrodes substrate) is up to 509.8 kPa^–1^ and the response and recovery times are 67.3 and 44.8 ms, respectively. After 10 000 loading–unloading cycles, the sensor still maintains excellent stability. The sensor also owns the self‐healing function. After the cutting–healing of the sensitive layer, the sensitivity reduction of the sensor is less than 16.2%. The tactile feedback is also achieved by connecting the sensor to a circuit. Overall, we fabricated a high‐performance pressure sensor by using a low‐cost and universal method. The sensor shows good self‐healing function and can be used for tactile feedback and it has potential application in robotics and electronic skin.

## 4 Experimental Section

### Preparation Process of the MXene (Ti_3_C_2_T*_x_*) Nanosheet Colloidal Solution

First, 0.50 g of precursor MAX phase (Ti_3_AlC_2_) was slowly added into the mixed solution of 0.50 g LiF and 10.0 mL 75% HCl, and the reaction was carried out with magnetic stirring at 35 °C for 24.0 h. Then the reactant was centrifugally washed to pH > 6.0, and the supernatant of the last centrifugation should be dark green, which indicated that MXene was successfully synthesized. Finally, the MXene was dispersed in a certain amount of deionized water and inert gas was introduced, along with an ultrasonic treatment at low temperature for 1.0 h. The MXene solution was centrifuged at 3500 rpm for 1.0 h after ultrasonic treatment, and the supernatant was collected as MXene (Ti_3_C_2_T*_x_*) nanosheet colloidal solution.

### Preparation of the Sensitive Layer with the Spinosum Structure

A mount of PU was cast on the surface of the abrasive paper mold with different roughness. The PU with spinosum structure was obtained by peeling dried PU from the surface of the abrasive paper. Then, the PU was placed on a heating table, and the MXene colloidal solution was uniformly sprayed on the surface of the microstructure until the square resistance of the surface is in the range of 5.0 to 30.0 Ω. Accordingly, a sensitive layer with spinosum structure can be obtained.

### Fabrication of the Interdigital Electrodes and Assembly of the Sensor

The interdigital electrode mask was fixed on an arbitrary flexible substrate with adhesive tape. Then the MXene colloidal solution was uniformly sprayed on the surface of the flexible substrate in the heating condition, and the heating temperature was set at 50–90 °C. The MXene‐based flexible interdigital electrodes can be obtained by peeling off the interdigital electrodes mask when the linear resistance per centimeter of the flexible substrate is lower than 5.0 Ω. Finally, the sensitive layer and the MXene‐based interdigital electrodes were placed face to face. The fully sprayed MXene‐based pressure sensor can be obtained by connecting two copper wires to the interdigital electrodes through the silver paste.

### Characterizations and Measurements

The morphology and thickness of the MXene nanosheets were characterized by TEM (FEI Titan G2 60‐300) and AFM (AIST‐NT). The MXene film was analyzed by multifunctional X‐ray diffractometer and Raman spectrometer (LabRAM HR Evo, 532.0 nm laser). The spinosum structures on the surface of the sensitive layers were characterized and measured by cold field emission SEM (JSM‐6700F) and the step profiler.

### The Sensing Performance Test of the Sensor

The sensor performance test system includes a uniaxial motor, a dynamometer, and a source meter (Agilent B2091A), all of which are connected and controlled by a computer. Specifically, the electrodes of the pressure sensor are connected with a source meter and a driving voltage of 0.10 V is supplied to the sensor through the source meter. Then, the sensor is clamped between the dynamometer and the uniaxial motor, and the uniaxial motor is controlled by a computer to squeeze the sensor at different pressures. Finally, the pressure and electrical output of the sensor are recorded by a computer.

## Conflict of Interest

The authors declare no conflict of interest.

## Supporting information

Supporting Information

Supplemental Video 1

Supplemental Video 2

## Data Availability

The data that support the findings of this study are available from the corresponding author upon reasonable request.
