# Supplementary material for: High‐Performance Flexible Pressure Sensor with a Self‐Healing Function for Tactile Feedback
Source: Adv Sci (Weinh). 2022 Apr 15;9(20):2200507. doi: 10.1002/advs.202200507 (PMC9284154; doi:10.1002/advs.202200507)
Supplement: Supplementary file 1 — Supporting Information [file ADVS-9-2200507-s003.pdf]

Supporting information

High-performance flexible pressure sensor with a self-healing function for tactile feedback

Mei Yang, Yongfa Cheng, Yang Yue, Yu Chen, Han Gao, Lei Li, Bin Cai, Weijie Liu*, Ziyu Wang*, Haizhong Guo*, Nishuang Liu, Yihua Gao

M. Yang, Y. Chen, H. Gao, L. Li, B. Cai, W. Liu, H. Guo
Key Laboratory of Material Physics, Ministry of Education, School of Physics and Microelectronics, Zhengzhou University
Zhengzhou 450052, P. R. China
Email: hguo@zzu.edu.cn; wjliu@zzu.edu.cn

Y. Cheng, N. Liu, Y. Gao
Center for Nanoscale Characterization & Devices (CNCD), School of Physics & Wuhan National Laboratory for Optoelectronics (WNLO), Huazhong University of Science and Technology (HUST)
Wuhan 430074, P. R. China

Y. Yue
Information Materials and Intelligent Sensing Laboratory of Anhui Province, key Laboratory of Structure and Functional Regulation of Hybrid Materials of Ministry of Education, Institutes of Physical Science and Information Technology, Anhui University
Hefei 230601, P. R. China

Z. Wang

The Institute of Technological Sciences, Wuhan University

Wuhan 430072, P. R. China

E-mail: zywang@whu.edu.cn

H. Guo

Collaborative Innovation Center of Light Manipulations and Applications, Shandong Normal University

Jinan 250358, P. R. China

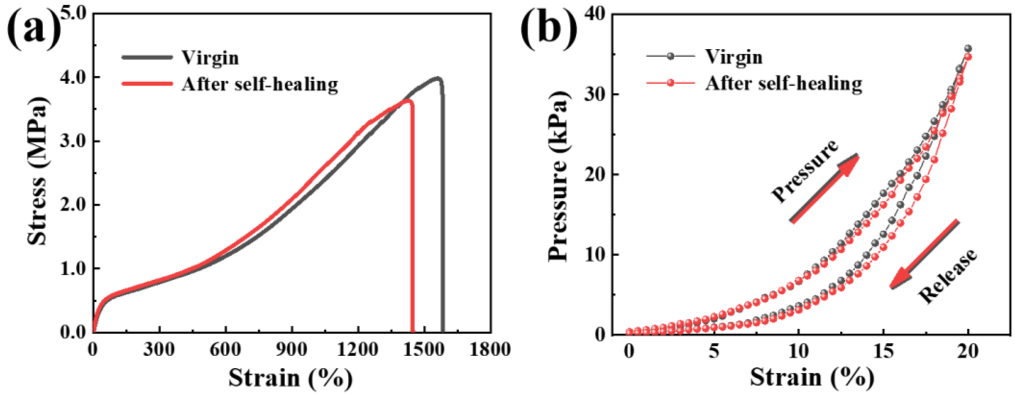

Figure S1. Stretchability and compressive strength tests on PU before and after self-healing. a) The stress-strain curve and b) the pressure-strain curve.

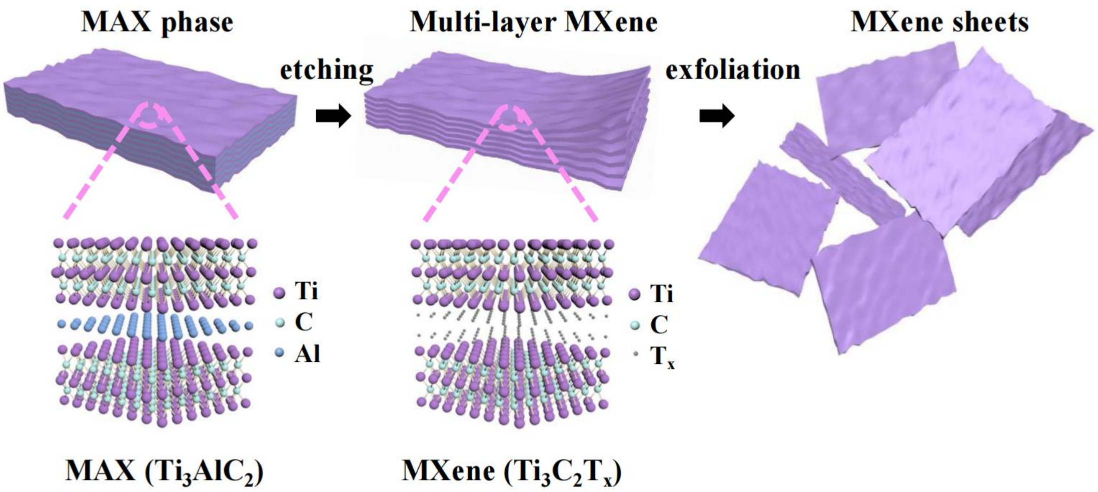

Figure S2. The preparation of MXene ($Ti_3C_2T_x$) from the MAX phase (Ti_3AlC_2).

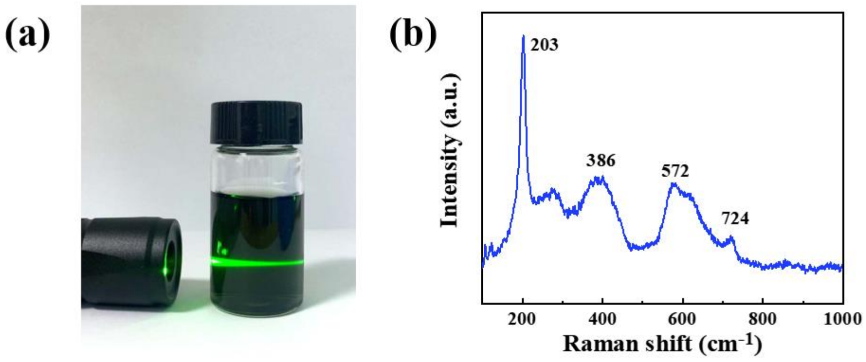

Figure S3. (a) Tyndall effect of the MXene solution. (b)Raman spectrum of the MXene film.

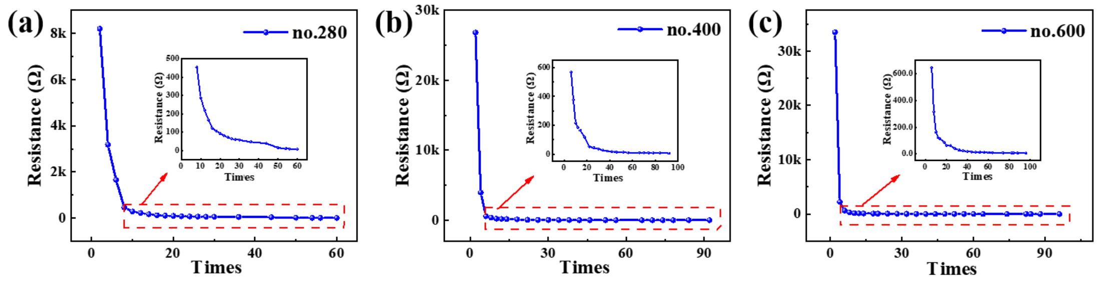

Figure S4. The time-dependent square resistance of the (a) no.280, (b) no.400, and (c) no.600 microstructure sensitive layer.

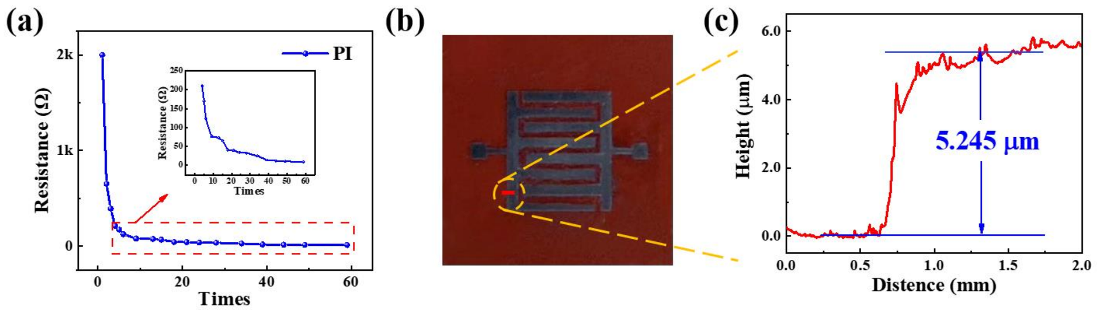

Figure S5. Characterization of the MXene-based interdigital electrodes (PI as flexible substrate). (a) The time-dependent line resistance of the MXene-based interdigital electrodes. (b) Optical image of the MXene-based interdigital electrodes. (c) The thickness of the MXene-based interdigital electrodes.

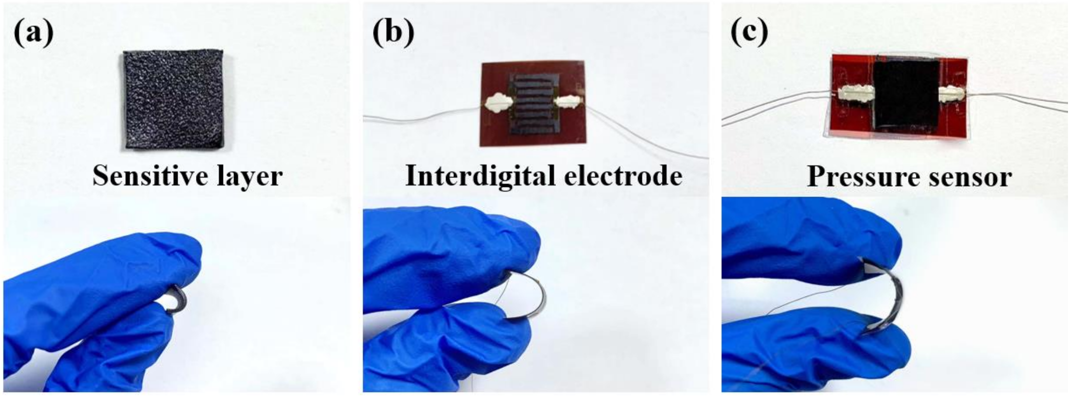

Figure S6. The optical images and bending tests of (a) MXene/PU sensitive layer, (b) MXene-based interdigital electrodes (PI as flexible substrate), and (c) MXene-based flexible pressure sensor.

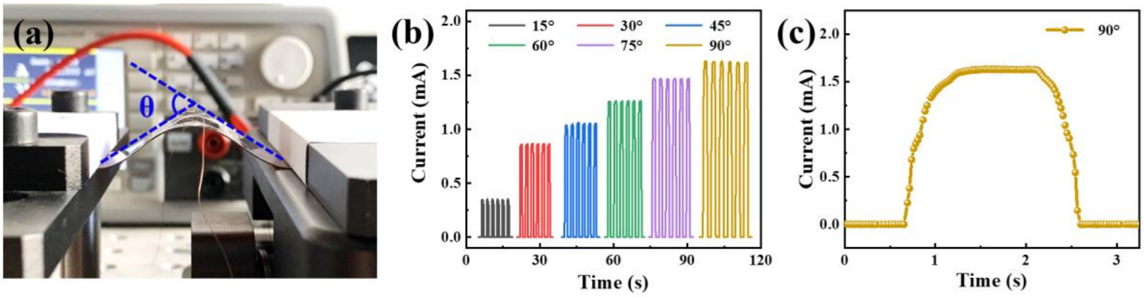

Figure S7. The sensing performance of the sensor in different bending states. a) Test system. b) I-T curves of the no.280 sensor at different bending angles. c) I-T curve of the no.280 sensor stretching between 0° and 90°.

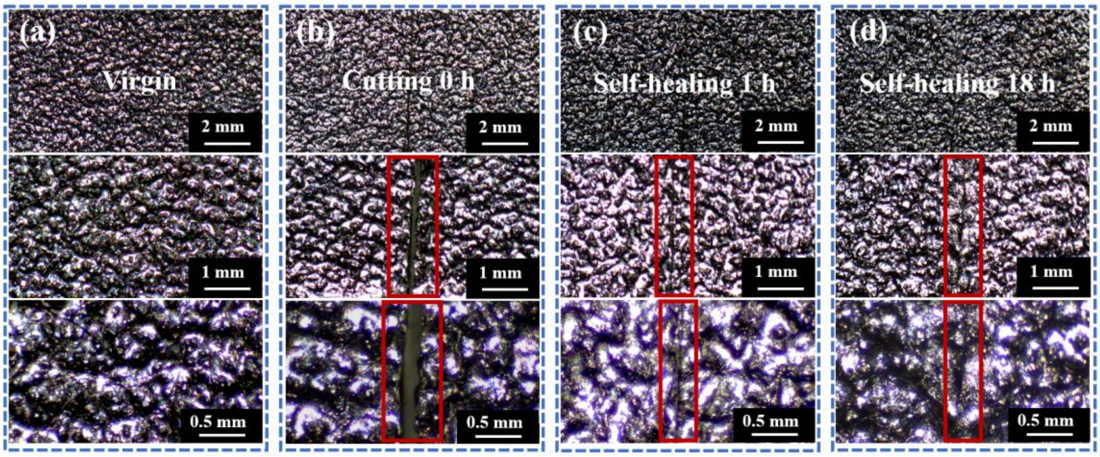

Figure S8. The cutting-healing process of the sensitive layer. The optical images of the no.280 sensitive layer at the different times: (a) virgin, (b) just after cutting, (c) self-healing 1 h, and (d) self-healing 18 h.

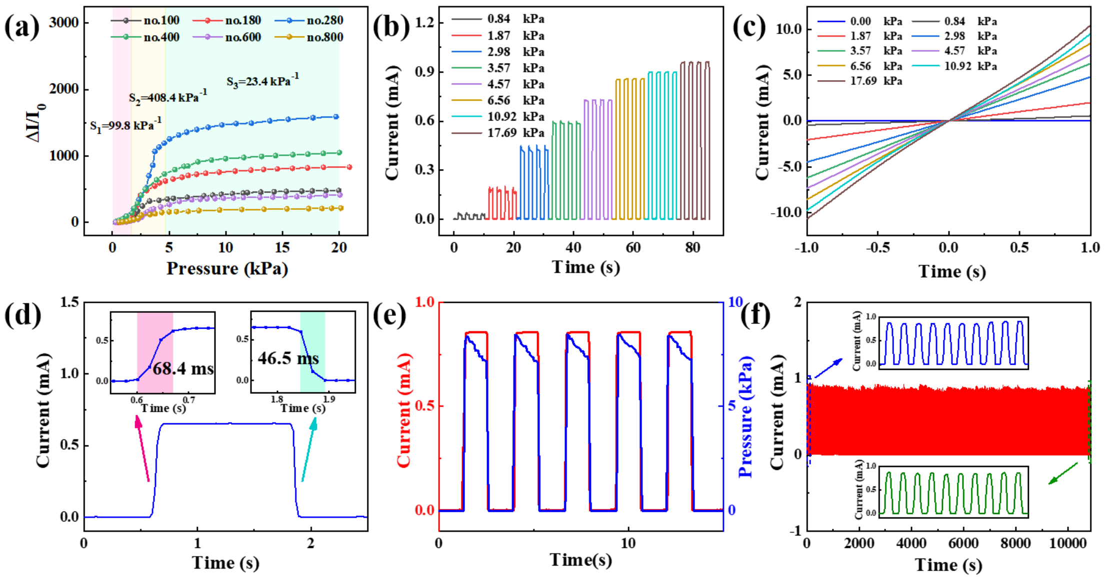

Figure S9. Sensing properties of the MXene-based pressure Sensor (The membrane filter as the interdigital electrodes substrate). (a) Sensitivity of the no.100-no.800 sensor. (b) The I-T curves of the no.280 sensor under serial pressures. (c) The I-V curves of the no.280 sensor under serial pressures. (d) The response and recovery time of the sensor. (e) The response of I-T and P-T curves under periodic loading-unloading. (f) After 10,000 pressure cycles at 10.7 kPa, the device shows excellent stability.

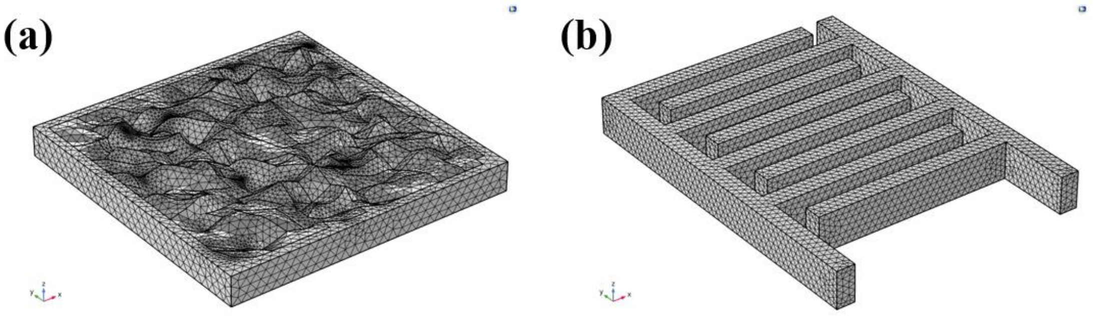

Figure S10. Meshing graphs of simulation models of (a) the MXene/PU sensitive layer and (b) the MXene-based interdigital electrode.

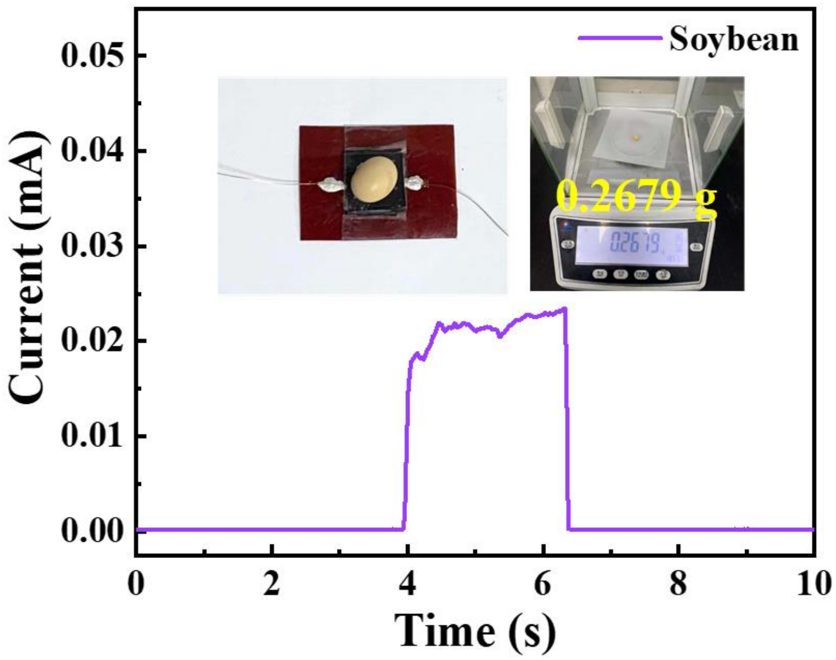

Figure S11. The tiny pressure exerted by a soybean (26.3 Pa).

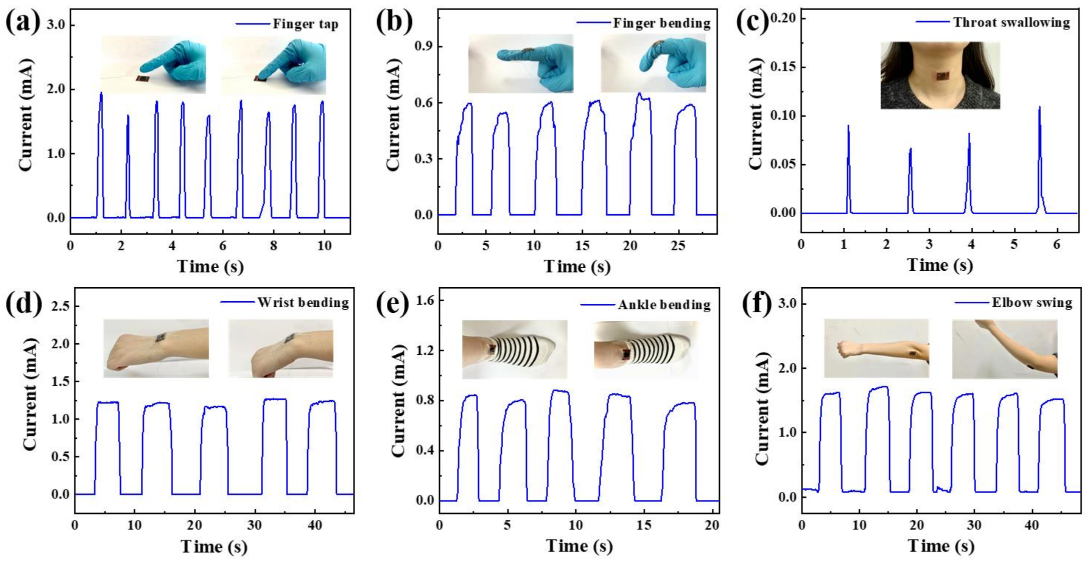

Figure S12. The real-time monitoring of human activity by the self-healing sensor. The signal responses in the form of current changes come from a) finger tap, b) finger bending, c) throat swallowing, d) wrist bending, e) ankle bending, f) elbow swing.

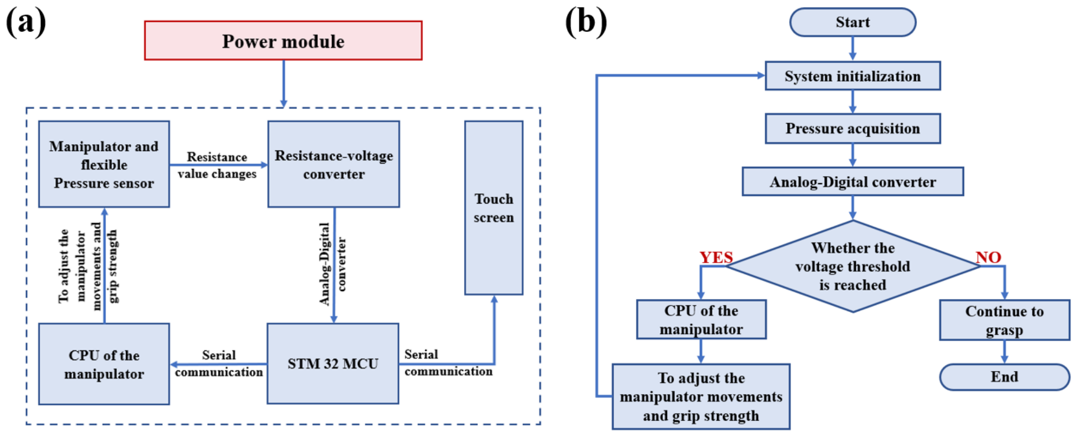

Figure S13. (a) Hardware structure and (b) software flow diagram of tactile feedback system.

Table S1. Performance comparison of different sensors.

Mechaniam	Materials	Detection limit (Pa)	Maximum sening range (kPa)	Sensitivity (kPa^{-1})/ΔP (kPa)	Response time (ms)	Recovery time (ms)	Cycles	Reference
Pizoresistive	MXene/tissue paper	10.2	30	0.55/ (0.023-3.036),<br>3.81/ (3.036-10)	11	25	10000	[1]
Pizoresistive	MXene/PDMS	4.4	15	151.4/ (0.0044-4.7),<br>33.8/ (4.7-15)	125	104	10000	[2]
Pizoresistive	SF@MXene	100	20	25.5/ (0.1-0.5),<br>3.4/ (1-20)	40	35	3500	[3]
Pizoresistive	MXene/NMC	8	7	24.63/ (0.01-0.2),<br>1.18/ (0.2-7)	14	16	5000	[4]
Pizoresistive	PET&Au&MXene	9	4.5	99.5/ (0.05-1),<br>4.0/ (1-4.5)	4	13	10000	[5]
Pizoresistive	SWNT-MXene	13	10	11.47/ (0.013-0.77),<br>0.23/ (2-10)	50	20	10000	[6]
Pizoresistive	MXene/Textile	-	40	3.844/ (<29),<br>12.05/ (29-40)	26	50	5600	[7]
Pizoresistive	MXene/PU	7.8	20.3	281.54/ (0.2-1.7),<br>509.78/ (1.7-5.7),<br>66.68/ (5.7-20.3)	67.3	44.8	10000	This work

Table S2. The data of the no.100-800 sensors before and after cutting-healing.

	**virgin**		**after self-healing**		**reduction**
	ΔP (kPa)	S (kPa^{-1})	ΔP (kPa)	S (kPa^{-1})	percent
**no.100**	0.4-3.4	118.51	0.4-4.1	115.75	2.33%
	3.4-19.9	28.34	4.1-20.3	27.47	3.07%
**no.180**	0.5-6.5	183.82	0.3-6.7	165.68	9.87%
	6.5-20.3	14.82	6.7-20.4	13.59	8.30%
**no.280**	0.2-1.7	281.54	0.3-2.0	281.43	0.04%
	1.7-5.7	509.78	2.0-5.7	456.87	10.38%
	5.7-20.3	66.68	5.7-20.7	57.08	14.40%
**no.400**	0.2-7.9	295.80	0.2-7.6	261.80	11.49%
	7.9-19.9	33.36	7.6-20.7	27.98	16.13%
**no.600**	0.2- 6.5	218.55	0.3-7.0	203.03	7.10%
	6.5-19.5	18.84	7.0-19.3	18.70	0.74%
**no.800**	0.4-5.5	17.30	0.4-5.9	17.22	0.46%
	5.5-19.8	3.02	5.9-20.9	2.94	2.65%

References

[1] Y. Guo, M. Zhong, Z. Fang, P. Wan, G. Yu, *Nano Lett.* **2019**, *19*, 1143.

[2] Y. Cheng, Y. Ma, L. Li, M. Zhu, Y. Yue, W. Liu, L. Wang, S. Jia, C. Li, T. Qi, J. Wang, Y. Gao, *ACS Nano* **2020**, *14*, 2145.

[3] D. Wang, L. Wang, Z. Lou, Y. Zheng, K. Wang, L. Zhao, W. Han, K. Jiang, G. Shen, *Nano Energy* **2020**, *78*, 105252.

[4] K. Wang, Z. Lou, L. Wang, L. Zhao, S. Zhao, D. Wang, W. Han, K. Jiang, G. Shen, *ACS Nano* **2019**, *13*, 9139.

[5] Y. Gao, C. Yan, H. Huang, T. Yang, G. Tian, D. Xiong, N. Chen, X. Chu, S. Zhong, W. Deng, Y. Fang, W. Yang, *Adv. Funct. Mater.* **2020**, *30*, 1909603.

[6] Y. Zhang, T.-H. Chang, L. Jing, K. Li, H. Yang, P.-Y. Chen, *Adv. Mater. Interfaces* **2020**, *12*, 8392.

[7] T. Li, L. Chen, X. Yang, X. Chen, Z. Zhang, T. Zhao, X. Li, J. Zhang, *J. Mater. Chem. C* **2019**, *7*, 1022.
